# Measures and Their Equivalents

**Published:** 1876-11

**Authors:** 


Measures and their Equivalents.—Our friends of the *American Grocer* recently published an article stating the death of a child from an overdose of opium, which was contained in a prescription administered in "teaspoonful" doses. Investigation showed that the family had used a large size teaspoon, and had thereby given three-sixteenths instead of one-sixteenth of a certain preparation of opium. The writer, in commenting on the case, says: "The many accidents that formerly ocurred in the putting up of medical prescriptions, led to the passage of a State law requiring every prescription clerk to pass a severe examination, and requiring him to possess a diploma based on such examination. In this law, one of the most important safeguards seems to have been left out. As most of the liquid medicines are administered in doses of "teaspoonfuls," the law should require the druggist to state plainly on the label what constitutes a 'teaspoonful' in medical parlance."

We thank our contemporary for the idea suggested, and, while we do not think that legislation is exactly necessary, to cover such cases, we believe that physicians should advise their patients more explicitly regarding the administration of their remedies. Especially in the case of children, medicines should be prescribed by drops, as the most reliable and efficient means of determining the exact dose.

The following table designates the equivalent of miscellaneous measures:

| | |
|---|---|
| Teaspoonful . . . . . | about 1 fld drachm. |
| Dessert " . . . . . | " 2 " " |
| Table " . . . . . . | " 4 " " |
| Wineglassful, . . . | " 2 " ounces. |

| | | | | |
|---|---|---|---|---|
| Teacupful, | " | 4 | " | " |
| Breakfastcupful, | " | 8 | " | " |
| Tumblerful, | " | 8 | " | " |
| Thimbleful, | " | $\frac{3}{4}$ | " | drachm. |
| Pinch (of leaves or flowers) | " | 1 | drachm | (Troy). |
| Handful, | " | 10 | " | " |

—*Druggists' Advertiser.*

